# The interplay between the immune response and neoadjuvant therapy in breast cancer

**DOI:** 10.3389/fonc.2025.1469982

**Published:** 2025-05-12

**Authors:** Noémie Thomas, Theodoros Foukakis, Karen Willard-Gallo

**Affiliations:** ^1^ Molecular Immunology Unit, Institut Jules Bordet, Brussel, Belgium; ^2^ Translational Breast Cancer Research, Department of Oncology-Pathology, Karolinska Institute, Stokholm, Sweden

**Keywords:** breast cancer, neoadjuvant treatment, immune microenvironment, tumor infiltrating lymphocytes, immune response, immune biomarker

## Abstract

Treatment of early breast cancer is currently experiencing a rapid evolution because of important insight into tumor subtypes and continuous development and improvement of novel therapeutics. Historically considered non-immunogenic, breast cancer has seen a paradigm shift with increased understanding of immune microenvironment, which have revealed extensive heterogeneity in tumor-associated inflammation. Notably, the more aggressive breast cancer subtypes, including triple-negative and HER2-positive, have exhibited favorable responses to combined chemo-immunotherapy protocols. Neoadjuvant therapy has emerged as the standard of care for these tumors, with pathological complete response used as a surrogate endpoint for long-term clinical outcomes and coincidently expediting new drug approval. The neoadjuvant setting affords a unique opportunity for *in vivo* treatment response evaluation and effects on the tumor microenvironment. In this review, the predictive and prognostic value of the tumor immune microenvironment before, during, and after treatment across various therapeutic regimens, tailored to distinct breast cancer subtypes, is carefully examined.

## 1 Introduction

In the 1990s, it was recognized that administration of systemic chemotherapy before surgery could downstage locally advanced, large breast cancer (BC). This allowed more patients to have breast conserving surgery thereby reducing the physical and psychological impact (1). Consequently, researchers determined that neoadjuvant systemic therapy (NAT) administered in BC patients was just as effective as standard adjuvant treatments (2–4). Guidelines were set for neoadjuvant treatment of all stage II-III BC together with other stages of the aggressive triple negative (TN) and human epidermal growth factor receptor (HER2)-positive subtypes and is still routine clinical practice until this date (5, 6). Research providing greater insight into BC biology guided the development of drugs specifically targeting the individual BC subtypes. Identification and characterization of surgical samples from poor NAT responders also fostered the development of new neoadjuvant and adjuvant therapies, thereby improving patient outcomes compared with adjuvant treatments in the TN and HER2+ BC subtypes (7). With the current speed of new drug development, there is a pressing need for trials designed to efficiently evaluate new drug combinations (8, 9). In addition, the collection of tumor specimens should no longer be limited to just pre- and post-treatment but also include on treatment samples. This interim analysis can provide more insight into the heterogeneity in response and can guide treatment adaptations.

Recent cumulative data have clearly established a key role for the immune system in cancer development and response to treatment. Immune infiltration (generally described as TIL for tumor infiltrating lymphocytes or leukocytes) in BC is very heterogeneous, with higher TIL densities found more frequently in the higher grade TN and HER2+ subtypes. These high-grade tumors are characterized by greater genomic instability and tumor mutational burden, potentially boosting tumor-specific neoantigen frequencies (10, 11). While some studies show that somatic mutations can drive anti-tumor immunity (12), others reported an inverse relationship between genomic heterogeneity and immune infiltration in TNBC, raising questions about the importance of TIL subpopulation balances in the tumor microenvironment (13–15). Some drugs used to treat BC patients increase tumor immunogenicity with preclinical studies, suggesting that cytotoxic agents partially exert their anti-tumor activity by inducing immune responses specific to tumor cells. Analyses of anthracycline-mediated immunogenic cell death found changes in cell surface molecule composition and soluble mediator release with some of the latter known to promote dendritic cell (DC) maturation and immune activation (16). Others detected direct immunomodulatory effects by these cytotoxic agents (17). Targeted treatments, including anti-HER2 antibodies and anti-estrogen therapies, have also been shown to elicit immunomodulatory effects (18).

Chemotherapy is the backbone of neoadjuvant treatment for most BC subtypes, with studies showing that a multidrug approach combining chemotherapeutic compounds leads to better outcomes (19). Immunotherapy as a monotherapy for BC was found inadequate, leading to trials adding it to conventional chemotherapy in efforts to increase response rates. Currently, the most widely used and best-known immunotherapies are monoclonal antibodies targeting immune checkpoint molecules, with multiple clinical trials investigating the addition of programmed cell death protein 1 (PD-1) or programmed death-ligand 1 (PD-L1) blockade. The standard of care for TNBC in the neoadjuvant setting (American Society of Clinical Oncology, ASCO guidelines) is now chemotherapy plus Pembrolizumab (20). Other immune checkpoint blocking antibodies also elicit anti-tumor activity, exemplified by anti-cytotoxic T-lymphocyte associated protein 4 (CTLA-4), which is less widely used due to its toxic side effects. The targeting of immune molecules like lymphocyte-activation gene 3 (LAG3), indoleamine 2,3-dioxygenase (IDO), V-domain Ig suppressor of T cell activation (VISTA), OX-40, and 4-1BB, have not yet been studied in combination with NAT. Meanwhile, other approaches with the potential to enhance anti-tumor immunity in the neoadjuvant setting are still in the early stages for BC (21, 22).

Treating patients in the neoadjuvant setting provides unique opportunities to analyze heterogeneity in the tumor and its microenvironment. Technological advances in genomics and proteomics that allow the simultaneous analysis of many molecules in small tissue specimens has accelerated our understanding of the tumor microenvironment (23), with further development of these techniques in the spatial context progressing rapidly (24). These new approaches have the power to address important research questions using limited clinical samples, such as a longitudinal analysis of biopsies at baseline, on treatment, and post-treatment; however, they require meticulous logistics to ensure high-quality sample collection and data analysis performed by a team of clinicians, scientists, bioinformaticians, and other specialists.

## 2 The pre-treatment tumor immune microenvironment

**Figure 1:**
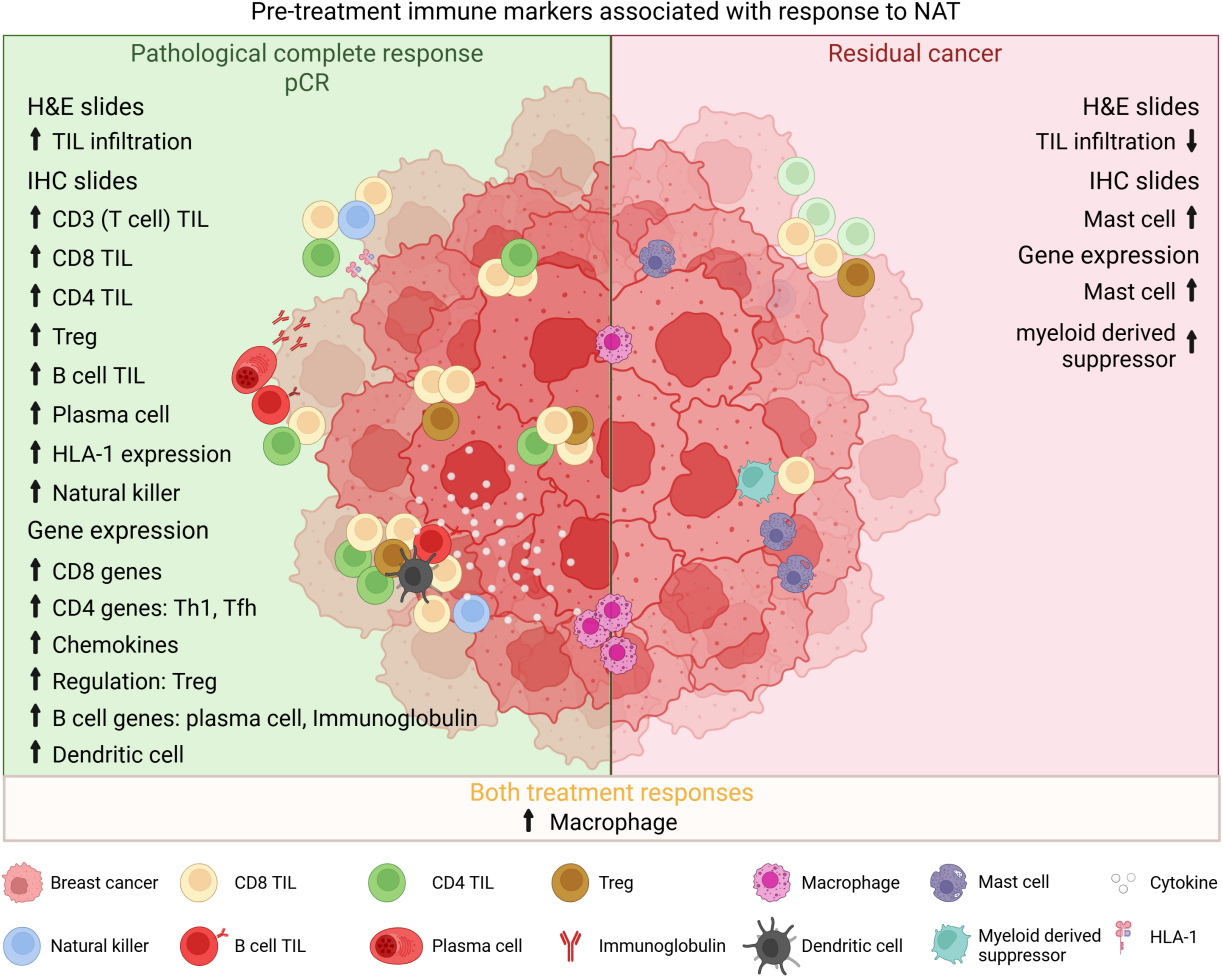
Interplay between pre-treatment TIME and treatment response. Overview of the most consistent interactions found between pre-treatment infiltrating immune cells and response to NAT. H&E, hematoxylin-eosin; TIL, tumor-infiltrating lymphocytes; IHC, immunohistochemistry; Treg, regulatory T cell; HLA, human leucocyte antigen; Th, helper T cell; Tfh, follicular helper T cell.

A patient’s preexisting tumor immune microenvironment (TIME) sets the stage, in part, for neoadjuvant therapy responsiveness. In recent years, broad analysis and characterization of TIL in pre-treatment biopsies using various technical approaches have been published. Hematoxylin and eosin (H&E) staining is the most common, inexpensive, and simple technique employed in the routine pathology lab. Semi-quantitative scoring on H&E-stained tumor sections has been developed and now put into place for routine assessment of stromal TIL in BC, following guidelines from the International Immuno-Oncology Biomarker Working Group on Breast Cancer (https://www.tilsinbreastcancer.org/) (25). TIL are, however, a collection of various cellular subpopulations, some with clear anti-tumor capacities, such as cytotoxic effector functions, while others play pro-tumor roles by promoting a TIME that supports tumor cell growth (26). Technological advances have improved analysis of the various immune cell subpopulations and their activation/functional profiles. Initially, chromogenic immunohistochemistry (cIHC) was used to stain specific immune subpopulations with more recent advancements in multiplex IHC (mIHC) driving this specificity even further (27). In parallel, next-generation sequencing and data analysis have produced increasingly refined immune gene signatures that identify critical pathways and inferred cell types in breast tumors and their TIME (28). Currently, the primary focus has been on quantifying immune cells and biomarkers associated with the adaptive immune system because of their known role in antigen-specific anti-tumor immunity. Recent evidence also advocates an important role for innate immunity in the response to treatment via key interactions with tumor and other immune cells through secretion of cytokines and chemokines and interactions with strategic cell surface proteins (29). **Figure 1** summarizes the most consistent interactions between pre-treatment immune cell infiltration and the response to NAT in BC.

### 2.1 Tumor-infiltrating lymphocytes

The notion that a preexisting immune activity in BC is associated with better clinical outcomes was first seen in neoadjuvant-treated patients. Incremental increases in TIL scores paralleled higher pathological complete response rates post-NAT (30). A subsequent large pooled analysis of six neoadjuvant trials confirmed the predictive value of TIL scoring for all BC patients (31). This analysis also revealed that pretreatment TIL scores were prognostic for long-term positive clinical outcomes in TN and HER2+ BC but associated with poorer outcomes for hormone receptor (HR)-positive luminal BC patients. Translational studies of TNBC patients treated with a combination of chemotherapy and immunotherapy determined that stromal TIL were associated with pathological complete response (pCR) but not predictive for benefit from immunotherapy (32–35). High TIL scores were also predictive for pCR in HER2+ patients treated with a combination of chemotherapy plus an anti-HER2 antibody or other HER2-targeted drugs (36).

Additionally, TIL were predictive for responses to other anthracycline-free neoadjuvant chemotherapy regimens (37, 38). In the TBCRC 006 trial, where patients were treated with a combination of Herceptin and Lapatinib without chemotherapy, those achieving a pCR had higher TIL, although this did not attain significance (39). Interestingly, in a subgroup of these patients characterized by high TIL, pCR was linked to higher CD4 T cell and CD20 B cell (TIL-B) TIL. In the PerELISA trial, in HER2+HR+ patients receiving endocrine therapy, TIL percentages were not associated with response (40). The correlation between TIL and anti-proliferative responses following neoadjuvant endocrine therapy is contradictory, with high stromal TIL associated with a poor response in a study by Dunbier et al. (41), while patients with low TIL achieved a >50% decrease in Ki-67 in Dieci et al. (42).

It has been suggested that scoring methodology for the neoadjuvant setting should be modified to include TIL distribution. One study scored TIL by volume, taking into account the percentage of stroma and found a more significant association for pCR and overall survival (OS) compared with classical TIL scoring in TNBC (43). The indication of a diffuse TIL pattern should be considered based on its superior association with pCR and improved prognosis (44).

### 2.2 Adaptive TIL subpopulations in the TIME

All patients with high TIL do not have positive responses, suggesting that specific cellular phenotypes may be more predictive for neoadjuvant treatment (45). A notable CD3 (all T cells) TIL presence has been reproducibly associated with anti-tumor immune responses, particularly if characterized by a significant CD8 T cell TIL presence coupled with markers of active cytotoxicity (46). CD4 TIL also make major contributions to anti-tumor immunity by helping and directing CD8 TIL and TIL-B responses (47). Higher pCR rates following NAT have been associated with CD8 and, to a lesser extent, CD3 or CD4 TIL (48–55). BC subtype analysis by Seo et al. (56) found the strongest association for TNBC, while only CD8 TIL were significantly associated with pCR for non-TNBC patients. Evaluation of pre-treatment samples from the GepraTrio trial revealed that, after CD3 TIL, TIL-B were most linked with pCR (30). TIL-B are more infrequent in the TIME; however, the significance of their presence and anti-tumor activity is now widely accepted (57). In the neoadjuvant setting, Brown et al. (58) showed that TIL-B cells were significantly associated with pCR in multivariate analysis, in contrast to T-cell TIL (CD3 or CD8). A well-known function of B cells is antibody secretion by differentiated plasma cells (PC), with these antibodies shown to have clinically relevant activities. Sakaguchi et al. (59) found a significant increase in CD79a/CD38 PC in BC patients achieving a pCR and HR- with a better disease-free survival (DFS).

Regulatory T cells (Treg; FoxP3), well-known and important regulators of immune responses via their suppressive actions, have also unexpectedly been associated with pCR (48, 55, 56, 60). Active immune responses are typically complemented by parallel increases in regulatory cells. Their balance, represented by the CD8:FoxP3 TIL ratio, has been significantly associated with pCR in various studies (61–63). Asano et al. (61), investigating BC subtypes, did not find significance for HR+ patients, but for TNBC patients, this ratio was significantly associated with pCR and prognostic for DFS and OS by multivariate analysis. On the other hand, analysis of pre-treatment biopsies from the NSABP-B27 trial identified a cluster of TIL associated with pCR that expressed high levels of CD4, CD20, CD68, FoxP3, and low CD8 (64). Recently, Abdelrahman et al. (65) found that low Treg TIL were associated with a good response in a small TNBC cohort.

The inclusion of Nivolumab in the Giada trial did not influence the predictive value of TIL subpopulations with CD4, CD8, and Treg all significantly associated with pCR (34), similar to previous studies. An immune cluster with high CD4, CD8, and TIL-B was more significantly associated with pCR than TIL scores in HER2+ BC patients treated with a combination of Lapatinib and Trastuzumab (39). Their detailed characterization of the immune microenvironment in patients treated with neoadjuvant chemotherapy-free regimens is uncommon.

An important understudied aspect of TIL is whether or not subpopulations and/or their frequencies are anti-tumor antigen specific, with newer biomarker studies focusing on the generation, presentation, and recognition of tumor-derived neoantigens. De Groot et al. (66) found that human leukocyte antigens (HLA) class I overexpression on tumor cells was significantly associated with (intracellular antigen presentation restricted) CD8 TIL and pCR. HLA-I expression, TIL infiltration, and positive treatment responses were also linked in HR+/HER2− BC patients from the GeparTrio trial (67), although high expression was associated with poor DFS. Yam et al. (68) went further by scoring CD3, CD8, and PD1+ TIL and performing T-cell receptor (TCR) sequencing on pre-treatment samples, finding that higher TCR clonality was significantly associated with increased CD3 and CD8 TIL and pCR. Interestingly, CD3 and CD8 TIL were located in close proximity to tumor cells in pCR patients. Their anti-tumor TCR specificity was not analyzed, but their data suggest that pre-treatment TIL are presented antigen and their presence in the TIME is associated with positive treatment-responses.

### 2.3 Innate TIL subpopulations in the TIME

Infiltration of innate immune cells in BC has sporadically been studied, particularly in the neoadjuvant setting. Increases in natural killer (NK;CD56) TIL within the TIME were associated with pCR and prognostic for DFS in HER2+ patients (69, 70). Tumors with intermediate HLA-I staining were characterized by increased NK TIL in association with pCR and DFS. Alternatively, in a study by Reddy et al. (71) mast cells were significantly lower in inflammatory BC patients with good treatment responses and had fewer interactions with CD8 TIL, M2 macrophages, and tumor cells.

Generally, macrophage and neutrophil infiltration of the TIME is associated with more aggressive tumors and poorer outcomes; although when also scoring tumor-associated macrophages (TAM; CD68), McLemore et al. (72) found that a higher ratio of TAM: TIL was a predictor of pCR following neoadjuvant chemotherapy. Okcu et al. (73) recently demonstrated that high neutrophil is better predictive for response than TIL. In contrast, Mitrofanova et al. (74) showed that higher TAM were in correlation with the number of positive lymph nodes and characteristic of BC patients not achieving a pCR. Kaewkangsadan et al. (75) detected an association between increased M2-polarized macrophages (CD163), generally thought to be pro-tumor, and pCR. The presence of peri-tumoral M2 TAM was strongly associated with pCR in TNBC (76); however, this association was not observed in the PROMIX trial (HR+ patients only) (77). Analysis of biopsies from the neoadjuvant I-SPY 1 trial found that high numbers of proliferating cell nuclear antigen (PCNA)-positive TAM were associated with higher grade and HR negativity (78). PCNA+ TAM were not predictive for treatment responses and associated with worse DFS in patients with substantial residual disease. Interestingly, this population was also characterized by a higher expression of genes characterizing M1 TAM, often associated with anti-tumor immunity. These studies highlight the need for further analysis of the role TAM play in the response to neoadjuvant therapy.

### 2.4 Signatures of the pre-treatment TIME

In parallel to scoring TIL and quantifying immune cells in BC tissue sections, many studies have analyzed differential expression of immune gene transcripts in responders versus non-responders following neoadjuvant therapy. Immune metagenes and their related pathways have been used as predictive models for NAT responsiveness in TN, HER2+, and inflammatory BC.

#### 2.4.1 Adaptive immune gene signatures

Denkert et al. (30) first demonstrated the predictive value of immune genes in 2010, confirming their findings in the GerapSixto trial in 2015 (79). Subsequently, many prognostic immune gene signatures, including the “immunologic constant of rejection” (80), an eight-gene T-follicular helper (Tfh) signature with CXCL13 (81) that still holds true today for immunotherapy (82), immune gene clusters (83), and others, were all predictive for pCR (84–86). Some immune signatures were developed for prediction of responses to specific neoadjuvant treatments, including IRSN-23 (87) or the “inferred immune cell activity” (88). Although most predict responses irrespective of the subtype, there are important differences in treatment sensitivity and immunogenicity to consider between BC subtypes. A pooled analysis of publicly available gene sets from three neoadjuvant chemotherapy studies demonstrated that the value of immune signatures can vary depending upon the BC subtype (89). The immunity metagene by Hamy et al. (90) was correlated with response in all subtypes, but its performance was superior in the HER2+ group. In addition, the immune effective score was specifically predictive for responses in HER2+ patients (91). *In silico* analysis based on sequencing data (Cibersort) revealed differences associated with pCR between BC subtypes based on distinct immune signatures (92, 93). Single-cell sequencing identified distinct TIL-B subpopulations associated with tertiary lymphoid structures predictive for response (94). Analysis of the T- and B-cell receptor (BCR) repertoires revealed that low immunoglobulin (Ig) evenness, a measure of oligoclonal B-cell expansion, was correlated with an immune presence and predictive for pCR (95). A number of the abovementioned studies have correlated both TIL scores or IHC-stained immune cells and immune gene signatures to response, suggesting that the signatures also accurately reflect immune activities in the tumor. Many of these predictive signatures simulate immunological processes, including T-cell responses associated with CD8 genes, the Tfh metagene, Th1 signature (including IFNG and STAT1 genes), important chemokines [e.g., C-X-C motif chemokine ligand (CXCL) 9, CXCL10, CXCL11, and CXCL13), and B-cell responses leading to plasma cell differentiation, memory B-cell metagenes, and immunoglobulin (IGHG) production, and pathways (FOXP3, IDO1, CTLA4, and PDCD1 (PD-1)].

Limited data are currently available on associations between pre-treatment immune gene expression and long-term clinical outcome after NAT. Perez-Pena et al. (96) demonstrated that INFG, cytotoxicity, and Th1-related genes were significantly associated with 5-year DFS for TNBC patients. In the CALGB 40603 trial, a small number of immune-related (high Ig) genes were associated with pCR and DFS (95). Gene expression analysis in pretreatment biopsies from the NeoALTTO trial identified an association between DFS and STAT1, irrespective of pCR in HER2+ patients (97). In WSG-ADAPT HER2+ trial, several immune response signatures were correlated with both pCR and DFS irrespective of the treatment arm (98). Similarly, in the CALGB 40601 trial, five TIL-B/PC and Ig-related immune signatures were correlated short- and long-term outcomes (99). Importantly, in the CALGB trial, B-cell immune signatures were more associated with pCR and DFS in contrast to TIL scoring and T-cell signatures in the NeoALTTO trial (100, 101). Rediti et al. (102) found that BCR diversity in patients from the NeoALTTO and CALGB 40601 trials was associated with an immune response and is prognostic for clinical outcome, suggesting potential treatment de-escalation for patients with a high integrated score.

#### 2.4.2 Innate immune gene signatures

Less attention has been paid to innate immune genes, despite data showing that DC and M1 myeloid signatures are correlated with good responses while mast cell, M2, and myeloid-derived suppressor cell signatures reflect poor responses. An in-depth analysis of non-responders with high immune signature expression revealed upregulation of neutrophil-associated genes in TNBC (103). Sammut et al. (104) detected upregulation of Treg, NKdim, and M2 macrophage signatures in samples from non-responders who had high proliferation plus high immune scores. TNBC patients that were immune-rich but did not respond to chemotherapy plus Durvalumab had higher expression of neutrophil and macrophage chemoattractant in contrast to responders who were characterized by increased IFNG expression and activated T- and B-cell markers (105). In the I-SPY 2 trial, a myeloid diversity signature was associated with better responses to 2 of the 10 treatment arms but not to immunotherapy (106).

#### 2.4.3 Integrated immune gene signatures

Immune gene signatures can be strong, independent predictors for neoadjuvant treatment responses (84). In combination with additional gene signatures reflecting the activities of other key components, including tumor and stromal cells, the predictive value can increase (85). Stover et al. (107) found that proliferation signatures have the strongest predictive value for pCR with immune signatures coming in second, which is more applicable for HR+ than TNBC because the latter have other response signatures involved. Callari et al. (108) supported this by demonstrating that an immune metagene was significantly linked with pCR in TNBC and HER2+ but not HR+ patients where only a high proliferation/low-HR metagene was predictive. Recent results from the Brightness trial show that a combination of proliferation and immune activation signatures were the best predictors (109), which was corroborated by Sammut et al. (104). Finally, Prado-Vazquez et al. (110) showed that addition of an immune signature gave additional predictive value for luminal TNBC molecular subclassification.

Multi-omic approaches are being utilized with increasing frequency for clinical research to include tumor mutational burden and genomic alterations with immune activities. The study by Sammut et al. (104) integrated ribonucleic acid (RNA), deoxyribonucleic acid (DNA), and digital pathology data with clinicopathological parameters to achieve significantly better predictive scores. Zhu et al. (111) similarly demonstrated improved prognostic and predictive values for integrated tumor mutational burden and CD8/M2 macrophage scores in TNBC. In HR−/HER2+, specific tumor cell clones were recently found to be associated with immunogenicity and good response to NAT (112). Using a proteogenomic model, Anurag et al. (113) identified biomarkers for anthracycline-free NAT associated with pCR and resistance mechanisms to better tailor TNBC treatment. Data from the ISPY2 trial illustrated how a combination of biomarkers can be employed to create a model for predicting responses to 10 different treatment regimens (114, 115). The future promises even more integrated analyses to refine and support both prognostic and predictive markers.

## 3 Early changes to the on-treatment TIME in the neoadjuvant setting

Growing evidence suggests that early changes to the TIME have more predictive power for treatment response than the pre-treatment state. Clinical trials integrating interim sample analyses, after one to a few cycles, provide the possibility for treatment reevaluation based on responsiveness (9). Park et al. (116) found that a TIL increase 3 weeks after the first NAT dose was more significantly associated with achieving pCR than baseline TIL. Interestingly, for patients with residual disease, a TIL increase was seen at interim analysis but only in TNBC. In parallel with TIL, “hot” immune tumors characterized by higher T, B, NK, and cytotoxicity signatures were significantly associated with treatment response at intermediate time points. In immune “cold” tumors, a mast cell signature was upregulated and reflected a lower likelihood of achieving pCR. In the PROMIX trial, higher immune signatures and TIL scores following two cycles of NAT were more associated with good responses compared with baseline values (77, 117). TNBC patients receiving neoadjuvant anthracycline-free therapy, who had on-treatment upregulation of immune cytotoxicity and regulation signatures, were significantly more likely to achieve a pCR (33). Parkes et al. (118) did not find significant changes in TIL or DNA-damage immune response signatures after three cycles of anthracyclines; however, immune signature negative patients did upregulate adaptive immune and NK genes along with interferon signaling and cytotoxicity signatures. Alternatively, Magbanua et al. (119) found overall downregulation of gene expression, including immune signaling pathways, in samples taken 24 h after the first dose of NAT, perhaps not unexpected in this short time frame.

Increases in intratumoral TIL in the post-immune checkpoint window were associated with pCR in the GeparNuevo trial (120). In the Keynote-173 trial, a single administration of Pembrolizumab induced higher TIL in patients achieving a pCR; however, pre-treatment TIL were more significantly associated with response (121). A combination of Atezolizumab with anti-HER2 treatment in the GO29381 trial was associated with increases in CD8 TIL together with PD-L1 expression in the center of the tumor bed (122). Furthermore, increases in cytotoxicity signatures along with NK, macrophage, Treg, and immune checkpoint signatures were detected, although they were not associated with treatment response. BC patients with pre-existing overexpression of PD-1 on CD4 and CD8 TIL showed clonal expansion after one dose of Pembrolizumab (123). This was associated with increases in TIL, Tfh, and “exhausted” CD8 cells, higher cytotoxic activities, and T helper 1 and IFNG signatures. Increases in PD-L1 expression on regulatory DC and specific macrophage subpopulations and HLA expression on tumor cells were also observed.

On-treatment TIL scoring in patient samples on anti-HER2 without chemotherapy have a high predictive value for pCR and were associated with long-term positive clinical outcome (124, 125). In parallel, expression profiling found significant upregulation of adaptive and innate immune genes at day 8 after HER-2 treatment in patients that go on to achieve a pCR (126). Varadan et al. (127) found that one cycle of Trastuzumab upregulated an immune index signature, which then became predictive for treatment response compared to baseline. Hurvitz et al. (128) observed similar immune signature increases after 2-3 weeks of anti-HER2 treatment, but the immune and TIL scores were not significantly associated with responsiveness. The same observations were made in the on-treatment samples from the Kristine trial, irrespective of treatment group (129). Endocrine treatment ± anti-cyclin-dependent kinase (CDK) 4/6 inhibitors in the NeoMonarch trial did not change TIL scores after 2 weeks of dual inhibition, but increases in adaptive immune and cytokine signaling signatures were detected (130). In the CORALLEEN trial, TIL scores did not change during anti-CDK4/6 treatment, but immune gene expression were decrease in contrast to chemotherapy (131). Finally, Dunbier et al. (41) did not find any significant alterations in immune genes/pathways after 2 weeks of Anastrozole.

## 4 Prognostic value of the post-treatment TIME

### 4.1 TIL scoring

NAT-induced changes in the TIME after NAT remains to be clarified due to very contradictory findings. Pelekanou et al. (132) found no significant changes in stromal TIL, but TIL increases were associated with better DFS. In contrast TIL decreases were observed following treatment in the SWOG S0800 trial, with treatment-induced immune changes uncorrelated with pCR or survival (133). Similarly, a decrease in stromal TIL was detected by Watanabe et al. (134), with higher post-treatment TIL significantly associated with an increased risk of recurrence in HR+ BC. An overall decrease in stromal TIL was observed in HER2+ BC, with higher post-treatment stromal TIL detected in large residual tumors and associated with worse outcomes (135, 136). In TNBC, increases and decreases in stromal TIL both were associated with better DFS (137). Conflicting findings for TIL after endocrine treatment have also been reported, such as increases in CARMINA02 trial responders (>50% decrease in tumor burden) (138), while increases in the DBCG trial were associated with poor responses (<30% decrease in tumor cells) (139).

Post-NAT TIL scoring in large TNBC cohorts with residual disease revealed a better prognosis was associated with high stromal and intratumoral TIL, particularly for patients with a high tumor burden (140–143). Elevated post-treatment stromal TIL were also associated with better DFS in HER2+ BC (144). One study further showed that adding TIL to the Residual Cancer Burden (RCB) produced a better prognostic factor than either alone across all BC subtypes (145). Thus, TIL scores plus RCB class in the post-NAT setting could better guide adjuvant treatment choices (146).

### 4.2 TIL subpopulations

Similar to global TIL, current data on TIL subpopulation changes following NAT is inconsistent. Urueña et al. (147) found a trend towards increased CD4, CD8, and TIL-B but not Treg or macrophages in the TIME after treatment. Dieci et al. (148) also found increases in CD8 TIL in TNBC residual disease. Gazinska et al. (149) observed that next to increased CD4 and CD8 cells, a significant rise in NK cells occurred in TNBC but not luminal BC, while in both subtypes, TIL-B were significantly decreased. Ladoire et al. (49) detected increases in CD8 and decreases in Treg TIL, reflecting an increased CD8:FoxP3 ratio, characterizing pCR patients. They subsequently found that this ratio was significantly associated with DFS and OS in a larger cohort of patients, including those not achieving a pCR (150). These findings were confirmed by Goto et al. (151). Kuroda et al. (152) reported that in addition to high post-treatment CD8 and low Treg, high TIL-B were associated with positive long-term outcomes for patients with TNBC. Conversely, Lejeune et al. (153) recently demonstrated that high CD4 and FoxP3, associated with CXCL13 expression and DC infiltration, are predictive for DFS in non-pCR TNBC. García-Martínez et al. (64) found that decreases in CD4 cell, TIL-B, and macrophage densities were significantly higher in pCR patients. Interestingly, for their patients with residual disease, increases in CD8 TIL and low total TIL post-treatment were associated with better clinical outcomes. Similarly, Reddy et al. (71) observed T-cell and TIL-B decreases, significant for CD8 TIL, and increases in Treg associated with a pCR. Post-treatment samples from these pCR patients revealed significant decreases in mast cells associated with increased M2 macrophages. In residual tumors from TNBC, T-cell Ig- and mucin-domain-containing molecule-3 (TIM-3) expressing M2 (CD163) macrophages were associated with better DFS (154). Kaewkangsadan et al. (75) observed decreases in infiltrating DC, but this was not associated with a response. Changes in the post-treatment TIME may reflect the state of the immune response at surgery, with the time after patients cleared their tumors (pCR) affecting the activity of remaining TIL. In patients with residual disease, this could signal the breadth and depth of immune responses induced by NAT, which may or may not have ended with a lower residual tumor burden had the time to surgery been deferred.

CD4 and Treg TIL upregulation in non-responders was thought to reflect an immune suppressive TIME in HER2+ patients treated with chemotherapy plus Trastuzumab and Pertuzumab (136). Alternatively, addition of anti-HER2 treatment increased T, Treg, TIL-B, and NK TIL, and granzyme B-positive TIL, particularly in good response patients, when compared to chemotherapy alone (155). This suggests that important additional immune-activating effects are elicited by these antibody drugs (156). Changing the administration to subcutaneous injection of anti-HER2 molecules was demonstrated to be even more immunogenic (157). Ladoire et al. (158) observed that T-helper TIL (Tbet+) were significantly induced by taxane-Trastuzumab treatment and prognostic for long-term clinical outcome. In HR+ patients, endocrine treatment increased the CD8:FoxP3 ratio in responders identified as having a 40% reduction in their Ki-67 index, suggesting an immunomodulatory effect (159). Oner et al. (160) associated overall increases in CD4 TIL with a good response, while Fukui et al. (161) showed that increased global TIL and FoxP3 cells characterize poor responders.

### 4.3 Immune signatures

A comparison of immune gene expression in pre- vs post-NAT samples revealed an overall decrease in immune-related signatures, which was most pronounced in TNBC (116, 162). Global decreases in the number of specific clonotypes was detected by Park et al. (163), with the biggest pre- vs. post-treatment changes in pCR patients. These data suggest that expansion of tumor-specific clonotypes occurs during treatment with neoadjuvant chemotherapy. This change in TCR and BCR repertoire was not confirmed in HER2+ patients treated with anthracycline-free NAT (164). In concordance with the TIL phenotypes, in the study by Gazinska et al. (149), post-NAT residual tumors are immune cold with an important decrease in TIL-B gene signatures but an increase in, predominantly immature non-functional or exhausted, NK TIL. Overall decreases in TIL gene expression were confirmed by Axelrod et al. (165), but unlike previous TNBC studies, increases in T- and NK-cell activation signatures were associated with improved DFS and OS. In Araujo et al. (166), post-treatment TNBC samples revealed that next to TIL scoring, higher C-C motif chemokine ligand (CCL5) expression was well correlated with better long-term clinical outcomes. Increased expression of immune genes in residual TNBC was also significantly associated with lower relapse rates with an eight-gene signature predicting relapse in the whole cohort and RCB subgroups (167). In HR+ patients treated with NAT single-cell sequencing revealed a relative increase in TIL with more proliferative CD4, cytotoxic CD8, and macrophages in the chemo-sensitive group (168).

Results from the Katherine study found no changes in immune expression between pre- versus post-NAT+anti-HER2 treatment samples (169). High expression of immune checkpoint inhibitor signatures in residual tumors were associated with improved long-term outcome after adjuvant Trastuzumab. The response to adjuvant Trastuzumab Emtansine (T-DM1) was shown to be independent of residual immune activation. Expression analysis of post-menopausal women treated with neoadjuvant Anastrozol detected upregulation of genes associated with immune activation associated with the response to treatment (30% decrease in tumor volume) (138, 170). Bergiamo et al. (171) observed that changes in immune signatures were only detected in patients treated for more than 1 month. Interestingly, in the Neomonarch trial, most immune-related gene pathways decreased with anastrozole treatment alone, in contrast to the combination with Abemaciclib (130). There was a similar increase in immune gene signatures after Ribociclib and chemotherapy; interestingly, increased interferon-associated gene expression was associated with a continuing tumor cell proliferation (131).

## 5 Conclusions

Continuous research and development, together with increased frequencies of treatment in the neoadjuvant setting, have significantly improved BC clinical outcomes. A myriad of approaches designed to utilize the immune response to target tumors have yielded very favorable results for some patients. However, neoadjuvant treatment responses using pCR as the primary endpoint should not be over-interpreted, however, because this outcome does not always translate to improved OS (172, 173).

Drugs targeting the immune response, rather than tumor cells, are increasingly shown to be capable of generating more durable anti-tumor impact. These outcomes advocate for the importance of furthering our understanding of how immune cells interact with other cells, including cancer cells, in the tumor microenvironment. The beneficial effects of a patient’s natural immune response to their tumor detected at diagnosis is most notable in patients with aggressive BC subtypes when treated with chemotherapy, particularly anthracycline-based treatments. A parallel increased presence of anti-tumor cytotoxic cell activation and regulatory mechanisms was demonstrated to be predictive for treatment response. The association of regulatory cell with good clinical outcome was also described in primary BC but only in correlation with a high infiltration of cytotoxic cells (174). Making use of the activation/regulation ratio in patients with extensive TIL could provide greater insight for response to neoadjuvant treatment.

**Figure 2:**
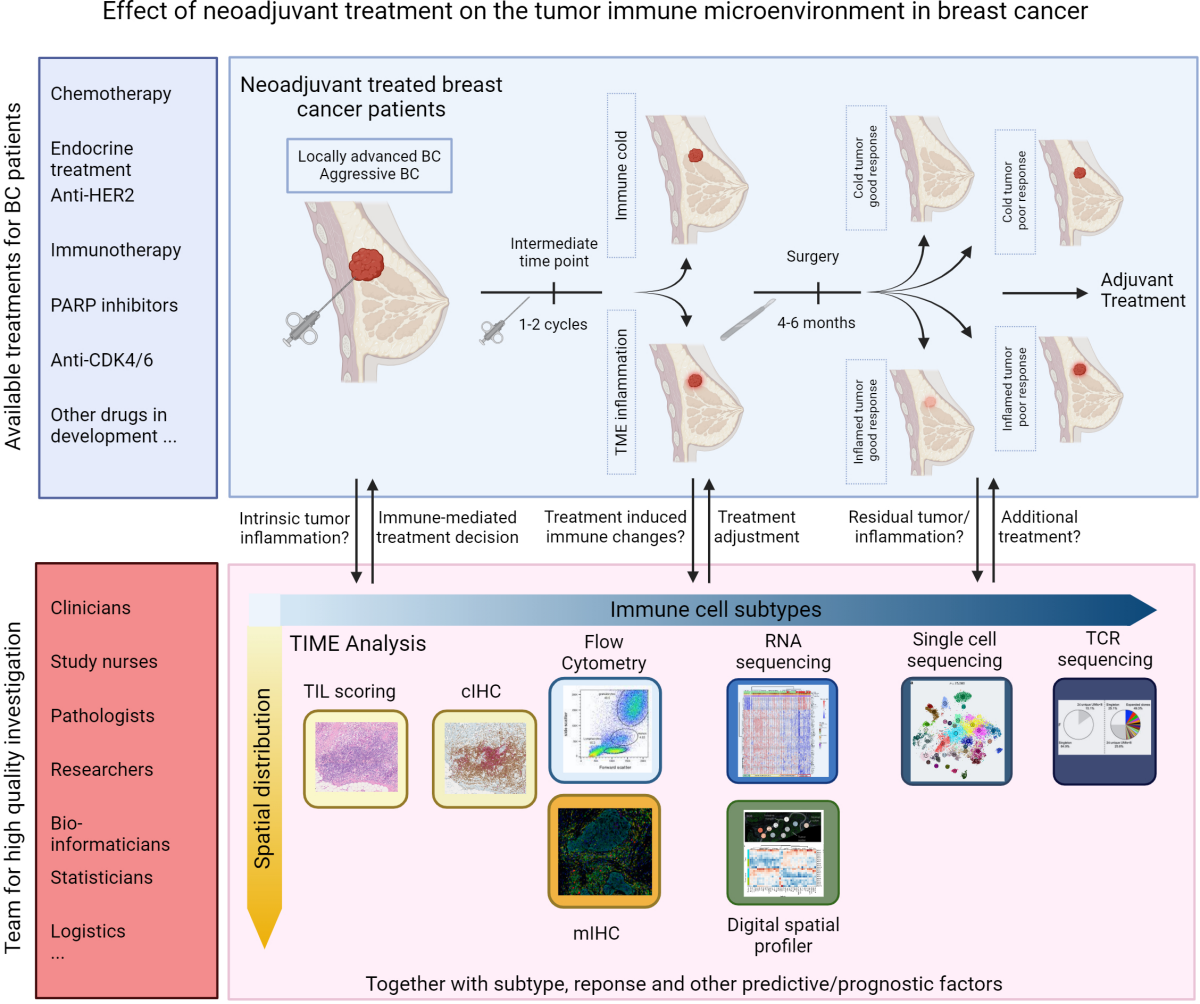
Opportunities for assessment of TIME in neoadjuvant trials in breast cancer.

Results from strategies designed to evoke anti-tumor immune responses in the immune-excluded or cold BC types using cancer vaccines, bispecific antibodies, and adoptive cell therapies have been modest at best (22). More detailed insight into the drivers of anti-tumor immunity have revived the development of new compounds that will potentially allow immunotherapy to become a cornerstone for treating all BC patients and facilitate treatment de-escalation with the omission of highly toxic chemotherapeutics. Tailoring the right treatment combination to individual patients will consequently become increasingly important. Conducting well-designed clinical studies that assess the tumor microenvironment at various time points using advanced technologies will significantly enhance our understanding of the antitumor potential of different treatments. **Figure 2** illustrates the available options for assessment of the TIME during neoadjuvant clinical trials in BC. Evaluating the immune responses of patients at intermediate time points on NAT could help to identify non-responders early on so that treatment is adjusted accordingly. Investigators should explore the use of window-of-opportunity trial designs to enable treatment modifications based on early tumor–immune responses. This approach will speed up the process of customizing treatment to the unique tumor characteristics of each patient. Post-treatment changes in tumor morphology and microenvironment composition are potentially confounding factors responsible for the observed variable results. Improving and establishing reproducible approaches for evaluating immune parameters in the post-treatment TIME should provide better clarity in the future.

Other than TIL scoring, there are no other currently validated methods for evaluating immune activities in the TIME, which may explain the conflicting data reported to date. Recent data suggest that the spatial distribution of immune phenotypes in the TIME may be even more informative than mere abundance (175, 176). Dontai et al. (177) found that the differential landscape between good and poor TNBC responders was primarily detected in the intra-tumoral infiltrating lymphocytes but not in the stroma. Next, Xu et al. (178) demonstrate that a cancer–T-cell interaction score is predictive for response to neoadjuvant immunotherapy. The rapid advancements in the field of spatial proteomics and transcriptomics will help uncover the spatial architecture of the TIME, which is crucial for comprehending its real intra-tumoral function. Integration of these immune parameters with other genomic markers should help to improve neoadjuvant treatment decisions for BC patients over the next decade.
